# TLR-2 and MyD88-Dependent Activation of MAPK and STAT Proteins Regulates Proinflammatory Cytokine Response and Immunity to Experimental *Trypanosoma congolense* Infection

**DOI:** 10.3389/fimmu.2019.02673

**Published:** 2019-11-22

**Authors:** Shiby Kuriakose, Chukwunonso Onyilagha, Rani Singh, Folayemi Olayinka-Adefemi, Ping Jia, Jude E. Uzonna

**Affiliations:** Department of Immunology, Rady Faculty of Health Sciences, Max Rady College of Medicine, University of Manitoba, Winnipeg, MB, Canada

**Keywords:** African trypanosome, immune response, proinflammatory cytokines, MAPK, STAT

## Abstract

It is known that *Trypanosoma congolense* infection in mice is associated with increased production of proinflammatory cytokines by macrophages and monocytes. However, the intracellular signaling pathways leading to the production of these cytokines still remain unknown. In this paper, we have investigated the innate receptors and intracellular signaling pathways that are associated with *T. congolense*-induced proinflammatory cytokine production in macrophages. We show that the production of IL-6, IL-12, and TNF-α by macrophages *in vitro* and *in vivo* following interaction with *T. congolense* is dependent on phosphorylation of mitogen-activated protein kinase (MAPK) including ERK, p38, JNK, and signal transducer and activation of transcription (STAT) proteins. Specific inhibition of MAPKs and STATs signaling pathways significantly inhibited *T. congolense*-induced production of proinflammatory cytokines in macrophages. We further show that *T. congolense-*induced proinflammatory cytokine production in macrophages is mediated via Toll-like receptor 2 (TLR2) and involves the adaptor molecule, MyD88. Deficiency of MyD88 and TLR2 leads to impaired cytokine production by macrophages *in vitro* and acute death of *T. congolense*-infected relatively resistant mice. Collectively, our results provide insight into *T. congolense*-induced activation of the immune system that leads to the production of proinflammatory cytokines and resistance to the infection.

## Introduction

African Trypanosomiasis, an infectious disease of humans and animals, is caused by various species of protozoan parasites belonging to the genus *Trypanosoma*. Because of minimal research in treatment and control measures, it is considered as one of the neglected tropical diseases similar to other parasitic diseases, such as Schistosomiasis, Leishmaniasis, Chagas disease, etc. (1). African animal trypanosomiasis is primarily caused by *Trypanosoma* (*T.) congolense, T. vivax, and T. brucei brucei* and presents as a mild disease in wild animals but very fatal in domestic animals if untreated. The disease has severe economic impact and adversely affects livestock production and farming in the affected regions of sub-Saharan Africa. *Trypanosoma congolense* is the most important African trypanosome and causes debilitating acute and chronic disease in cattle and other domestic animals. Because the parasites are purely extracellular but intravascular, they are unable to leave the circulation and are constantly exposed the to the host's immune system. As a result, they have developed sophisticated evasion mechanisms including antigenic variation of the variant surface glycoprotein (VSG) (2, 3), polyclonal B-lymphocyte activation (4), and induction of immunosuppression (5–7). Mice are the most common animal models for experimental African trypanosomiasis and have provided great insight into the immunopathogenesis of the disease. BALB/c mice are highly susceptible to experimental *T. congolense* infection because they are unable to control the first wave of parasitemia and die within 8–10 days. On the contrary, C57BL/6 mice are relatively resistant to infection and control several waves of parasitemia and survive for over 100 days (8). It has been shown that death of infected animals is due in part to hyper-activation of immune cells (particularly macrophages and T cells) resulting in excessive production of pro-inflammatory cytokines (including IFN-γ, IL-6, IL-12, and TNF), which leads to systemic inflammatory response like syndrome (8). However, the innate receptors, adaptor proteins and signaling pathways associated with *T. congolense-*induced cytokine production in macrophages are not known.

Toll like receptors (TLRs) are a family of type 1 trans-membrane receptors found on innate cells and play critical role in the initiation of innate immune response. Ligation of these receptors by their ligands leads to activation of various signal transduction pathways that lead to the induction of various genes including inflammatory cytokines. TLRs have an important role in recognition of molecular signatures of microbial infection, propagation of various signaling pathways, and directing the adaptive immune response (9). The extracellular leucine-rich repeats of TLRs are responsible for recognition of pathogens while the cytoplasmic region, known as Toll/interleukin-1 receptor (TIR) domains, are responsible for initiating intracellular signaling events (10). This involves heterophilic interactions of TIR domain with cytosolic adaptor proteins, such as myeloid differentiation of primary response protein 88 (MyD88), TIR-domain containing adapter protein (TIRAP), TIR domain-containing adapter inducing IFNβ (TRIF), and TRIF-related adaptor molecule (TRAM) (10, 11). However, MyD88 is the major adaptor protein that is a part of almost all TLR signaling pathways. Individual TLRs interact with different combinations of adaptor proteins resulting in the activation of various transcription factors, such as nuclear factor (NF)-κB, activating protein-1 (AP-1), and induces a specific immune response. Members of both mitogen-activated protein kinases (MAPKs) and signal transducer and activator of transcription (STAT) family members can interact to activate multiple intracellular signaling pathways that lead to increased cytokine production.

In this study, we investigated the innate receptors involved in *Trypanosoma congolense* recognition in macrophages, the role of MyD88, and the intracellular signaling molecules involved in *T. congolense-*induced cytokine production. We show that *T. congolense*-induced cytokine production is dependent on MyD88-mediated activation of MAPKs (ERK, p38, JNK) and STATs (STAT1 and STAT3) signaling and that TLR2 is the critical receptor involved in parasite recognition, production of proinflammatory cytokines, and induction of resistance in infected mice.

## Materials and Methods

### Mice

Six to eight weeks old female C57BL/6 and outbred Swiss white (CD1) mice used in this study were purchased from Charles River, St. Constante, Quebec, Canada. MyD88^−/−^, TLR2^−/−^, and TLR4^−/−^ mice were purchased from The Jacksons Laboratory (Bar Harbor, ME). Animals were housed at the Central Animal Care Services (CACS) facility, University of Manitoba, Winnipeg, Canada. The studies were approved by the University of Manitoba Animal Care Committee and carried out in accordance with the regulation of the Canadian Council on Animal Care.

### Reagents

Lipopolysaccharide (LPS) from *Escherichia coli* was purchased from DIFCO Laboratories (Detroit, MI). Rabbit anti-mouse p38 and ERK 1/2 mAbs, affinity-purified rabbit anti-phospho p-38, affinity purified mouse anti-phospho ERK 1/2, rabbit anti-total and phosphor-specific SAPK/JNK mAbs, rabbit polyclonal anti-STAT1, rabbit polyclonal anti-STAT3, and rabbit anti-phospho and total NF-κB mAb were purchased from Cell Signaling Technology (Danvers, MA). The p38 MAPK inhibitor 4-(4-Fluorophenyl)-2-(4-methylsulfinylphenyl)-5-(4-pyridyl)1H-imidazole (SB-203580), p42/44 ERK inhibitor 1,4-Diamino-2,3-dicyano-1,4-*bis*(2-aminophenylthio)butadiene (U-0126), and JNK inhibitor anthra[1,9-*cd*]pyrazol-6(2*H*)-one, 1,9-pyrazoloanthrone (SP 600125) were purchased from Calbiochem (Mississauga, Ontario, Canada). Fludarabine (specific inhibitor of STAT-1) was obtained from Sigma-Aldrich (Mississauga, Ontario, Canada). STAT3 inhibitor, 2-Hydroxy-4-(4-methylphenyl) sulfonyloxy)acetyl)amino)-benzoic acid (S31-201) was obtained from Santa Cruz Biotechnology (Dallas, TX).

### Infection and Estimation of Parasitemia

*Trypanosoma congolense* (Trans Mara Strain), variant antigenic type (VAT) TC13 was used in this study (12). Frozen TC13 stabilates were expanded in immunosuppressed (treated with cyclophosphamide) CD1 mice as previously described (12). After 3 days of infection, blood was collected from CD1 mice by cardiac puncture. Parasites were purified from blood using DEAE-cellulose anion-exchange chromatography (13), washed and resuspended in Tris-saline glucose (TSG) solution containing 10% heat-inactivated FBS (TSG-FBS) at a final concentration of 10^4^/ml. Mice (WT, MyD88^−/−^ and TLR2^−/−^) were infected by intraperitoneal injection of 100 μl TSG-FBS parasite suspension (containing 10^3^ parasites). Daily parasitemia was determined by counting the number of parasites in a drop of the blood using a microscope as previously described (14). Briefly, a drop of blood (taken from the tail vein of infected mice) on a microscopic slide was covered with a cover slip and the numbers of parasites present in at least 10 fields were counted at 400× magnification.

### Preparation of Trypanosomal Whole Cell Extract (WCE)

To prepare *T. congolense* whole cell extract (WCE), isolated parasites were resuspended in TSG at a final concentration of 10^8^/ml and then subjected to 3–5 sonication cycles (5 min per cycle). Thereafter, the sonicate was further subjected to freeze/thawing (at −80°C) up to about 8 cycles (30 min/cycle), aliquoted and stored at −80°C until used. Endotoxin level in WCE preparations was determined by the LAL kit (E-TOXATE, Sigma) according to the manufacturer's suggested protocol. Endotoxin level was <0.05 EU/ml.

### Cell Lines, Bone Marrow-Derived Macrophages (BMDM), and *in vitro* Cell Cultures

The origin of ANA-1 cells or retrovirus-immortalized bone marrow-derived macrophage cell lines from C57BL/6 mice has been described previously (15). The immortalized cell lines were grown in complete RPMI medium (RPMI 1640 medium supplemented with 10% FBS, 10 U/ml penicillin/streptomycin and 50 μM 2-mercaptoethanol). Primary bone marrow-derived macrophages were differentiated from marrow cells as previously described (16). Briefly, bone marrow cells were isolated from the femur and tibia of C57BL/6 mice and differentiated into macrophages using conditioned media (complete RPMI medium supplemented with 30% L929 cell culture supernatant). On the 7th day, the cells were harvested, washed, cultured in 24-well plates (1 ml/well) for 24 h in the presence or absence of WCE (1:10 ratio) or LPS (1 μg/ml) and the culture supernatant fluids were collected and stored at −80°C until used for cytokine ELISAs. Two million (2 × 10^6^) cells/ml were used for all the *in vitro* culture experiments. In some experiments, the cells were pretreated with SB-203580 (p38 inhibitor, 10 μM), U-0126 (ERK inhibitor, 10 μM), SP-600125 (JNK inhibitor, 50 nM), Fludarabine (STAT1 inhibitor, 10 μM) or S31-201 (STAT3 inhibitor, 10 μM) for 1 h before stimulation with WCE or LPS.

### Isolation of Peritoneal Macrophages

Groups of mice were inoculated with 100 μl of PBS containing 10^3^ live parasites or 10^7^ parasite equivalent of WCE. The mice were sacrificed at different time points (30 min to 24 h) post inoculation, and the peritoneal lavage fluid was collected as previously described (17). Macrophages from peritoneal lavage were lysed with NP40 lysis buffer and used for western blots.

### Western Blot

Assessment of MAPKs, STATs, and NFKB p65 phosphorylation were determined by Western blot, as previously described (18). Briefly, ANA-1 cells, peritoneal macrophages or fully differentiated BMDMs were serum starved in petri-plates for 24 h and inoculated with WCE or LPS. At indicated times, the cells were washed with ice cold PBS, total protein was extracted using NP40 lysis buffer supplemented with protease inhibitor cocktail (1 mM sodium orthovanadate and 1 mM phenylmethylsulfonyl fluoride). The lysates (10 μg) were resolved in 10% SDS-PAGE and transferred onto polyvinylidene difluoride (PVDF) membranes (Amersham Biosciences, Quebec, Canada).The membranes were blocked with 5% BSA in TBST for 2 h at room temperature. The membranes were then incubated overnight with specific polyclonal rabbit antibodies against phosphorylated mouse p38, ERK1/2, JNK, STAT1, STAT3, STAT5, and NFkB p65 subunit. Thereafter, the blots were washed with TBST (5 times) and incubated with goat anti-rabbit HRP-conjugated secondary antibody and the bands were revealed by enhanced chemiluminescence (ECL) (Amersham, GEHealthcare Bioaciences, PA) reagents. Thereafter, the blots were routinely stripped and re-probed with antibodies against total p38, ERK, JNK, STAT1, STAT3 that were used as loading controls. Densitometric analysis was performed and integrated density values were presented as the ratio of phosphorylated protein over total compared with media control.

### Preparation of Spleen and Liver Cells for Cytokine Analysis

At day 9 after infection, mice were sacrificed and blood were collected for serum. The spleens and livers were collected and processed into single-cell suspensions. Red blood cells in the spleen cells were routinely lysed with ACK lysis buffer, and the cells were washed with PBS and resuspended in in complete tissue culture medium (DMEM supplemented with 10% heat-inactivated fetal bovine serum, 2 mmol L-glutamine, 100 U/mL Penicillin, and 100 μg/ml streptomycin). The liver samples were perfused through the right ventricle with 10 ml of ice-cold-PBS and digested with collagenase-D (125 μg/mL) for 30 min at 37°C before being homogenized. The cells were passed through a 70 μm cell strainer (VWR, Mississauga, ON, Canada) and washed by spinning at 1,200 rpm for 5 min. Washed liver cells were resuspended in 40% percoll (Sigma) and carefully layered above 70% percoll and centrifuged at 750 × g for 20 min at 22°C. The interface containing lymphocytes was collected and washed with PBS. The lymphocytes were resuspended at final concentration of 4 × 10^6^/ml in complete tissue culture medium and cultured for 48 h. The culture supernatant fluids were collected and assayed for cytokines by ELISA.

### Cytokine ELISA

The levels of cytokines (IL-1β, IL-6, IL-12p40, IFN-γ, TNF, and MCP-1) in serum of infected mice or culture supernatant fluids from BMDMs and ANA-1 cells stimulated with WCE or LPS or cultures of spleen and liver cells were determined by ELISA using paired Abs and appropriate cytokine standards (eBioscience) according to the manufacturer's suggested protocols. In another set of experiments the cells were pretreated with SB-203580 (10 μM), U-0126 (10 μM), SP-600125 (50 nM) for 1 h before stimulation with *Trypanosoma* whole cell extract or LPS. Supernatants were collected after 24 h and assayed for IL-6 and IL-12p40.

### Statistical Analysis

Cytokine data are presented as mean ± SEM. Differences in cytokine production between groups were compared using two-tailed Student's *t-*test and two-way ANOVA. Significance was considered if *p* < 0.05. GraphPad Prism software was used to analyse the data.

## Results

### *Trypanosoma congolense* (TC) Induces Proinflammatory Cytokine Production in Macrophage Cell Lines and Bone Marrow-Derived Macrophages (BMDM)

**Figure 1:**
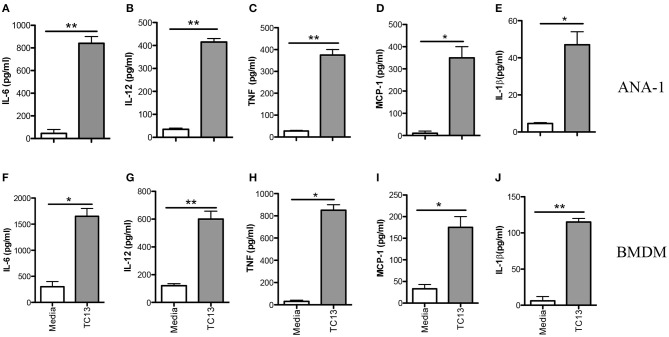
*Trypanosoma congolense* induces cytokine production in immortalized macrophage cell lines (ANA-1) and primary bone marrow-derived macrophages (BMDMs). ANA-1 cells and BMDMs were stimulated with *Trypanosoma congolense* whole cell extract (WCE, 1:10 ratio) and after 24 h, the culture supernatant fluids were collected and assayed for cytokines by sandwich ELISA. Shown are IL-6 **(A,F)**, IL-12p40 **(B,G)**, TNF **(C,H)**, MCP-1 **(D,I)**, and IL-1β **(E,J)** levels in the culture supernatant fluids of ANA cells **(A–E)** and BMDMs **(F–J)**. The data presented are representative of three independent experiments with similar results. ^*^p < 0.05; ^**^p < 0.01.

Infection of mice with African trypanosomes (including *T. congolense*) is associated with high levels of serum proinflammatory cytokines (including IL-12p40, IL-6, and TNF-α) and nitric oxide (NO) (19, 20). Indeed, death of *T. congolense-*infected mice is associated with cytokine storm leading to systemic inflammatory response like syndrome (SIRS) (8). However, the key innate immune cells and molecular mechanisms underlying recognition of *T. congolense* are not yet studied. Because macrophages play a central role in resistance to experimental African trypanosomiasis (21, 22), we investigated their role in proinflammatory cytokine production and the molecular pathways involved in this process. First, we stimulated immortalized macrophage cell line (ANA-1) with *Trypanosoma congolense* whole cell extract (WCE) for 24 h and assessed proinflammatory cytokine secretion by ELISA. WCE stimulation induced IL-6, IL-12p40, TNF-α, MCP-1, and IL-1β production in ANA-1 cells (Figures 1A–E). We also confirmed the induction of these cytokines in primary bone marrow-derived macrophages (BMDMs, Figures 1F–J). Collectively, these results show that *T. congolense* induces strong production of proinflammatory cytokines in macrophages.

### *T. congolense* Induces MAPKs and STATs Phosphorylation in Macrophages

**Figure 2:**
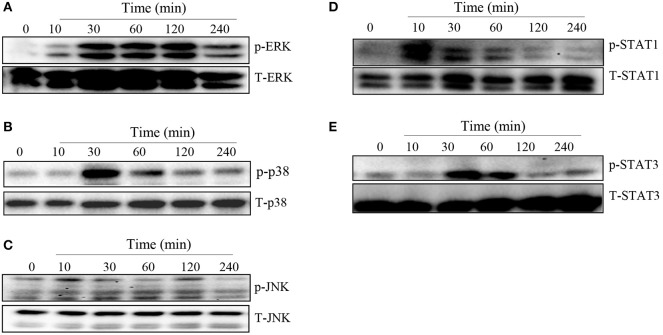
*T. congolense* induces phosphorylation of MAPKs and STATs in macrophages. BMDMs were stimulated with WCE and at the indicated times, the cells were lysed and the lysates were assessed by western blot for phosphorylation of ERK **(A)**, p38 **(B)**, JNK **(C)**, STAT1 **(D)**, and STAT3 **(E)** proteins using appropriate primary and secondary antibodies. The same blots were stripped and re-probed with antibodies against total ERK, p38, JNK, STAT1, and STAT3 and used as loading controls. The data presented is a representative of three independent experiments with similar results.

MAPK and STAT family of signaling molecules are important in regulating proinflammatory cytokine production in immune cells including macrophages (23). Although not yet demonstrated in *T. congolense* infection, several parasitic infections have been shown to activate MAPK and STAT signaling pathways in infected cells (24, 25). Therefore, we wished to determine whether the induction of proinflammatory cytokine production in macrophages by WCE involves activation of the MAPK and STAT pathways. We stimulated BMDMs with WCE and at different times, assessed phosphorylation of MAPK and STAT proteins by western blot. As shown in Figures 2A–E, WCE induced phosphorylation of ERK, p38, JNK, STAT1, and STAT3 proteins in primary BMDMs at various time points after stimulation. Collectively, these results suggest that *T. congolense*-induced cytokine production may be mediated through MAPK and STAT signaling pathways.

### Inhibitors of MAPKs and STATs Significantly Reduce *T. congolense*-Induced Cytokine Production in Macrophages

**Figure 3:**
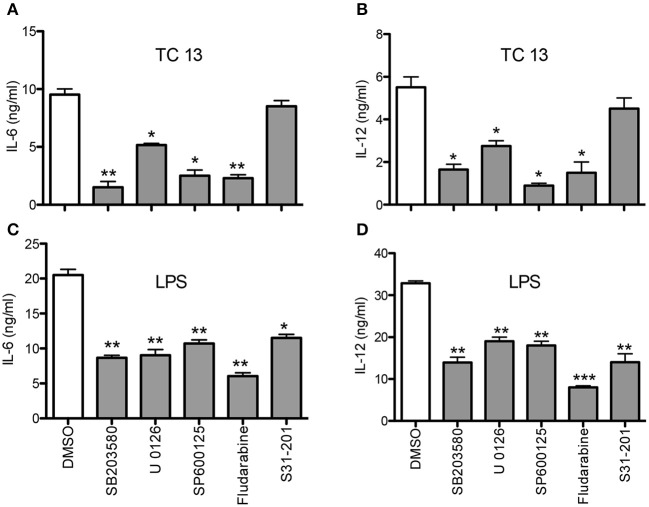
MAPK and STAT inhibitors abrogate *T. congolense*-induced IL-6 and IL-12p40 production in macrophages. BMDMs were treated with MAPK inhibitors (U0126 for ERK, SB203580 for p38, and SP600125 for JNK) and STAT inhibitors (Fludarabine for STAT1 and S31-201 for STAT3) for 1 h prior to stimulation with WCE **(A,B)** or LPS **(C,D)**. After 24 h, the levels of IL-6 **(A,C)** and IL-12p40 **(B,D)** in the culture supernatant fluids were determined by sandwich ELISA. The data presented are representative of three independent experiments with similar results. ^*^p < 0.05; ^**^p < 0.01; ^***^p < 0.001 compared to cells treated with DMSO (vehicle).

To directly determine the involvement of MAPKs and STATs in *T. congolense*-induced cytokine production in macrophages, we performed the above experiments in the presence or absence of specific inhibitors of p38 (SB203580), p42/p44 ERK (U0126), JNK (SP600125), STAT1 (Fludarabine), and STAT3 (S31-201). Pre-treatment of BMDMs with SB203580, U0126, SP600125, and Fludarabine before stimulation with *T. congolense* WCE significantly suppressed IL-6 and IL-12p40 production by these cells (Figures 3A,B). Interestingly, STAT3 inhibitor, S32-201, did not affect WCE-induced IL-6 and IL-12 production, indicating that WCE-induced STAT3 signaling is not as critical as MAPKs at inducing IL-6 and IL-12 production in macrophages. As positive controls, we showed that these specific inhibitors also blocked LPS-induced production of IL-6 and IL-12p40 in macrophages (Figures 3C,D). Taken together, these results confirm that members of MAPK (JNK, p38, ERK) and STAT1 play important role in controlling the intracellular events that lead to the production of IL-6 and IL-12p40 in *T. congolense* treated macrophages.

### *T. congolense* Induces MAPK and STAT Phosphorylation in Macrophages *in vivo*

**Figure 4:**
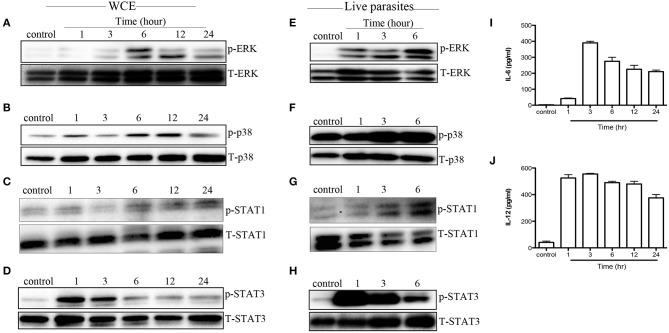
*T. congolense* induces MAPK and STAT phosphorylation in peritoneal macrophages. C57BL/6 mice were inoculated intraperitoneally with 100 μl WCE containing 10^7^ parasite equivalent **(A–D)** or 10^6^ live parasites **(E–H)**. At the indicated times, mice were sacrificed and peritoneal macrophages were isolated from peritoneal wash, lysed and assessed directly for phosphorylation of ERK **(A,E)**, p38 **(B,F)**, STAT1 **(C,G)**, and STAT3 **(D,H)** by western blot. In addition, the levels of IL-6 **(I)** and IL-12p40 **(J)** in the peritoneal lavage fluid of mice inoculated with WCE were also determined by ELISA. The data presented are representative of three **(A–D,I,J)** and two **(E–H)** independent experiments with similar results (*n* = 4–5 mice per each time point).

Next, we evaluated whether MAPK and STAT phosphorylation induced by WCE is reproducible *in vivo*. We injected C57BL/6 mice intraperitoneally with WCE and phosphorylation of MAPKs and STATs was assessed in the peritoneal macrophages directly *ex vivo*. As shown in Figures 4A–D, WCE injection significantly induced phosphorylation of ERK, p38, STAT1, and STAT3 in peritoneal macrophages. In another set of experiments, we injected live parasites into C57BL/6 mice i.p. and assessed MAPK and STAT phosphorylation in macrophages from the peritoneal lavage fluid at different times after infection. As with WCE, injection of live parasites also up-regulated the phosphorylation of MAPK and STATs (Figures 4E–H), and leads to a concomitant increase in IL-6 and IL-12 levels in the peritoneal lavage fluids (Figures 4I,J). These results show that *T. congolense*-induced phosphorylation of MAPK and STATs occurs following *in vivo* infection.

### MyD88 Is Involved in *T. congolense*-Induced Intracellular Signaling and Cytokine Production

**Figure 5:**
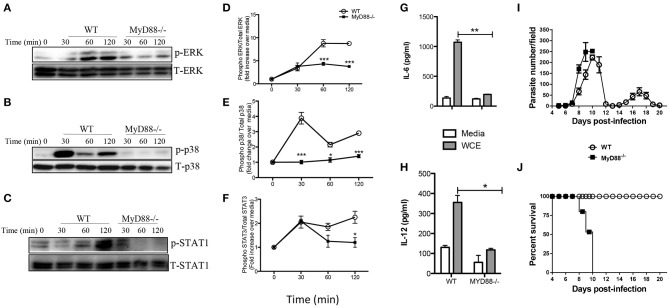
Intact MyD88 is critical for *T. congolense*-induced MAPK and STAT phosphorylation and resistance to infection. BMDMs from WT and MyD88^−/−^ mice were stimulated with WCE and at indicated times, the cells were lysed and the lysates were assessed by western blot for phosphorylation of ERK **(A,D)**, p38 **(B,E)**, and STAT1 **(C,F)** using appropriate primary and secondary antibodies. The same blots were stripped and re-probed with antibodies against total ERK, p38 and STAT1 and used as loading controls. The ratios of phosphorylated ERK **(D)**, p38 **(E)**, and STAT1 **(F)** to their respective total proteins were calculated by densitometry and plotted as line graphs **(D–F)**. In addition, the levels of IL-6 **(G)** and IL-12 **(H)** in the culture supernatant fluids were determined by ELISA. Groups (*n* = 6 per group) of MyD88^−/−^ and C57BL/6 (WT) mice were infected with 10^3^ *T. congolense* i.p and monitored daily for parasitemia **(I)** and survival **(J)** for about 21 days. The data presented are representative of two independent experiments with similar results. ^*^p < 0.05; ^**^p < 0.01; ^***^p < 0.001.

The central adaptor molecule, MyD88, plays a crucial role in initiating proinflammatory cytokine production in macrophages infected with other related protozoa including *T. cruzi* (26, 27) and *T. brucei* (28). To determine whether signaling via MyD88 is crucial for *T. congolense*-induced proinflammatory cytokine production, we stimulated BMDMs from MyD88^−/−^ and WT mice with WCE and performed western blot at different times to compare MAPK and STAT phosphorylation and production of proinflammatory cytokines. Deficiency of MyD88 completely abolished WCE-induced ERK, p38 and STAT1 phosphorylation in macrophages (Figures 5A–F) and this was associated with a concomitant inhibition of IL-6 and IL-12p40 production (Figures 5G,H).

Next, we investigated the effect of MyD88 deficiency in infection outcome by comparing parasitemia and survival period of *T. congolense*-infected WT and MyD88^−/−^ mice. Infected MyD88^−/−^ mice on the relatively resistant background developed higher and uncontrolled first wave parasitemia compared to WT mice and died within 10 days post-infection (Figures 5I,J). In contrast, all the infected WT mice controlled several waves of parasitemia and survived up to 20 days (when the experiment was terminated). The enhanced susceptibility of MyD88^−/−^ mice to *T. congolense* infection was associated with significant reduction in serum levels of IL-6, TNF-α, and IFN-γ (Figure S1). These results show that the central adaptor molecule, MyD88, is critical for resistance to *T. congolense* infection. They further suggest the involvement of TLRs in the recognition of trypanosomal molecule and subsequent initiation of innate immune response to the parasite.

### TLR-2 Is Essential for WCE-Induced MAPK and STAT Signaling and Resistance to *T. congolense* Infection Mice

**Figure 6:**
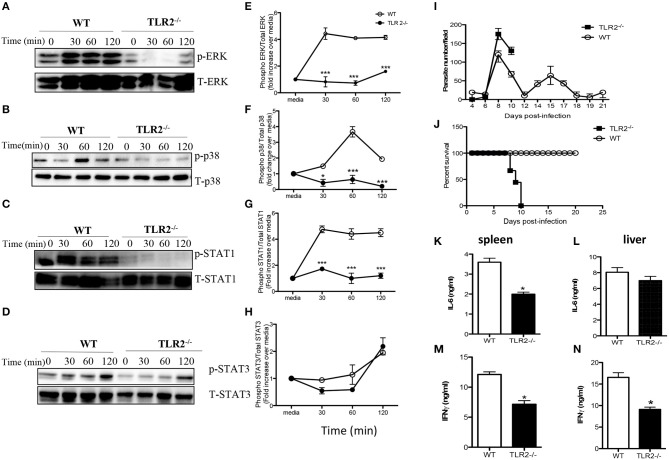
TLR2 is essential for *T. congolense*-induced MAPK and STAT phosphorylation and resistance to *T. congolense* infection. BMDMs from WT and TLR2^−/−^ mice were stimulated with WCE and at the indicated times, the cells were lysed and lysates were assessed by western blot for phosphorylation of ERK **(A)**, p38 **(B)**, STAT1 **(C)**, and STAT3 **(D)** using appropriate primary and secondary antibodies. The same blots were stripped and re-probed with antibodies against total ERK, p38, STAT1, and STAT3 and used as loading controls. The ratios of phosphorylated ERK **(E)**, p38 **(F)**, STAT1 **(G)**, and STAT3 **(H)** to their respective total proteins were calculated by densitometry and plotted as line graphs **(E–H)**. Groups (*n* = 12 per group) of TLR2^−/−^ and C57BL/6 (WT) mice were infected with 10^3^ *T. congolense* i.p and monitored daily for parasitemia **(I)** and survival **(J)** for about 21 days. At day 9 post-infection, some WT and TLR2^−/−^ mice were sacrificed and spleen and liver cells were cultured for 48 h and the production of IL-6 **(K,L)** and IFN-γ **(M,N)** were determined by ELISA. The data presented are representative of 2–3 independent experiments with similar results. ^*^p < 0.05; ^***^p < 0.001.

Toll like receptors (TLR) are important innate immune receptors involved in recognition of conserved molecular patterns expressed by microbes and the initiation of innate immune responses. Given that activation of MyD88 is usually associated with ligation of several TLRs, we sought to determine whether TLR signaling is involved in WCE recognition by macrophages. WCE-induced phosphorylation of ERK, p38 and STAT1 and the production of IL-6, IL-12p40, and TNF-α were not different in TLR4^−/−^ compared to WT macrophages (Figures S2A–I). In contrast, WCE-induced phosphorylation of p38, ERK, STAT1, and STAT3 was dramatically suppressed in TLR2^−/−^ macrophages (Figures 6A–H) and this was associated with dramatic reduction in IL-6, IL-12p40, and TNF-α production (**Figures S3A–C**).

Next, we investigated whether TLR2 plays a critical role in the outcome of infection by comparing parasitemia and survival period of *T. congolense*-infected WT and TLR2^−/−^ mice. Infected TLR2 deficient mice on the relatively resistant background developed higher and uncontrolled first wave parasitemia compared to WT mice and died within 10 days post-infection (Figures 6I,J). On the other hand, all infected WT mice controlled several parasitemia waves and survived up to 30 days that's when the experiment was terminated. The enhanced susceptibility of TLR2 deficient mice was associated with significant (*p* < 0.05) reduction in serum levels of IL-6, TNF-α, and IFN-γ (Figures S3D–F) and IL-6 and IFN-γ levels in their spleen and liver cell culture supernatant fluids, compared to their WT counterpart mice (Figures 6K–N). Interestingly, IL-10 levels in the serum (Figure S4A) and the production of IL-10 by spleen cells (Figure S4B) from infected TLR2^−/−^ mice were also significantly lower than those of their WT counterpart mice, suggesting that the lower levels of inflammatory cytokines in the serum of these mice may not due to dampening effects of IL-10 on their production. Collectively, these results show that TLR2 is a critical innate receptor involved in the recognition of *T. congolense* and initiation of optimal protective immunity to this parasite in mice.

## Discussion

Acute death of mice infected with African trypanosomes is usually associated with hyper-activation of immune cells (mostly macrophages and T cells), and excessive production of proinflammatory cytokines (including IL-6, IL-12p40, IFN-γ, and TNF) leading to systemic inflammatory response like syndrome (8). However, these cytokines are also important for efficient parasite control and resistance to African trypanosomes (29) suggesting that a fine balance must be maintained for survival. We previously showed that the production of proinflammatory cytokine by splenic and liver macrophages following *T. congolense* infection contributes to disease and treatment with Berenil suppressed (but do not completely abrogate) the production of these cytokines and prevents death in the highly susceptible mice (19). The primary focus of the present study was to investigate the innate receptor and signaling mechanisms involved in *T. congolense* recognition and induction of proinflammatory cytokine production in infected mice. We showed that *T. congolense* induces proinflammatory cytokine production in both primary and immortalized macrophages. We further showed that TLR2 is the innate receptor involved in *T. congolense* recognition by macrophages and this involves the central adaptor protein, MyD88. Furthermore, we showed that activation of MAPKs (p38, ERK, and JNK) and STATS (STAT1 and STAT3) occur in macrophages following recognition of *T. congolense* resulting proinflammatory cytokine production. Blockade of MAPKs and STATs with their specific inhibitors abrogated proinflammatory cytokine production. Collectively, these observations reveal the molecular and intracellular signaling events that lead to proinflammatory cytokine production in macrophages following their interaction with *T. congolense*.

TLRs are important for the recognition of microbes by innate immune cells (including macrophages) and initiation of innate immune response (30). Recent reports show that TLRs play an important role in initiating innate immune response against several parasitic infections including *T. brucei, T. cruzi, L. major*, and *Toxoplasma gondii* (26, 28, 31, 32). *T. cruzi*-derived glycophosphatidyl inositol (GPI) moiety that anchors the major surface glycoprotein to the cell membrane have been shown to trigger NFκβ activation and proinflammatory cytokine production in macrophages via TLR2 signaling (33). Indeed, we had previously shown that *T. congolense* induces NF-KB p65 phosphorylation in infected macrophages *in vitro* (34). In addition, DNA from *T. cruzi* stimulates cytokine production that is dependent on TLR9 and synergizes with parasite-derived GPI anchor for cytokine induction in macrophages (26). Another study showed that a subset of *T. cruzi* glycoinositolphospholipids stimulates cytokine production in macrophages via a TLR4-dependent and TLR2-independent manner (27). In contrast, *P. falciparum* GPI induces TNF production in macrophages by engaging several TLRs including TLR4, TLR2, and TLR1 (35, 36). Hitherto, no study has investigated the role of TLRs in *T. congolense*-mediated cytokine production. Our results show that the recognition of *T. congolense* leading to activation of intracellular signaling events and production of proinflammatory cytokines in macrophages is mediated through TLR2 and is independent of TLR4.

The binding of TLRs by their corresponding ligands leads to the recruitment of adaptor proteins and initiation of intracellular signaling events that ultimately result in proinflammatory cytokine gene expression. MyD88 is the most important adaptor molecule that is centrally involved in the activation of downstream signaling events following ligation of several TLRs by their respective ligands. The activation of MyD88 results in activation and/or phosphorylation of key signaling pathways including STATs and MAPKs. A recent report showed that deficiency of MyD88 leads to enhanced susceptibility to *T. brucei* infection, due in part to impaired IL-12 production by macrophages and a consequent impairment in Th1 response (28). In line with this, we found that deficiency of MyD88 leads to impaired phosphorylation of MAPKs and STATs in macrophages following *T. congolense* stimulation and this was associated with inability to control the first wave of parasitemia and death within 10 days in the relatively resistant mice. A recent report shows similar observation that the soluble variant surface glycoprotein (sVSG) of *T. brucei rhodesiense* initiates the expression of a number of proinflammatory genes including TNF, IL-12, IL-6, and iNOS by phosphorylation of ERK, p38, JNK, and NFκB pathways (37). Similarly, the GPI anchor of *T. cruzi* trypomastogotes has been shown to trigger phosphorylation of ERK and p38 in macrophages leading to NFκB activation and induction of pro-inflammatory cytokine genes (38). Furthermore, *T. gondii* has also been shown to induce MAPK and STAT3 phosphorylation in macrophages (25, 39). Thus, it appears that the activation of MAPKs and STATs may be a common pathway that is shared by protozoan parasites for the induction of inflammatory cytokines in macrophages.

TLRs recruit several TIR domain-containing adaptors, such as MyD88, TRIF, TIRAP/MAL, and TRAM for the induction of pro-inflammatory cytokine genes (40). MyD88 is recruited by extracellular TLRs, such as TLR2 and TLR4 and also associates with endosomal TLRs by binding to different lipids. However, TLR4-dependent activation of inflammatory genes occurs through MyD88 dependent and independent pathways (40–42). The MyD88-independent TLR4 signals occur through TRIF, an adaptor molecule that interacts with TRAF3 and TRF6 resulting in NF-κB and MAPKs activation and induction of several pro-inflammatory cytokines (40). Although not tested here, it is possible that TIRAP could indirectly contribute to proinflammatory cytokine gene activation and hence play a role in the overall immunity to *T. congolense* infection given that this adaptor molecule contributes to TLR2 signaling.

Although our *in vitro* and *in vivo* studies showed that TLR2 is critical for *T. congolense*-induced production of proinflammatory cytokines in macrophages, we found that deficiency of TLR2 (as seen in TLR2^−/−^ mice) results in increased susceptibility to the infection. TLR2^−/−^ mice on the usually resistant background were unable to control their first wave of parasitemia and succumbed acutely to the infection within 8–10 days post-infection. This finding suggests that TLR2 dependent immune activation plays a critical role in the overall immunity to *T. congolense* infection in mice. Thus, in macrophages (and perhaps other innate immune cells), TLR2 dependent recognition of *T. congolense* triggers cytokine responses that may be critical for initiating protective adaptive immunity necessary for effective parasitemia control. In line with this, we found that serum levels of IL-6, TNF-α, and IFN-γ in infected TLR2^−/−^ mice were lower than those in infected WT mice.

Although our study clearly showed the importance of TLR2 in proinflammatory cytokine production and resistance to *T. congolense* infection, the parasite molecule that is recognized by this innate immune receptor remains to be defined. Recent studies have shown that recognition of *T. cruzi* and *P. falciparum* GPI molecules by TLR2 on macrophages leads to activation and production of proinflammatory cytokines (26, 43, 44). Given that *T. congolense* variant surface glycoprotein is attached to the cell membrane via a GPI anchor, it is conceivable that *T. congolense* GPI-derived molecule may be the critical ligand for TLR2 in our system. In line with this, we observed that *T. congolense*-induced MAPK and STAT phosphorylation and production of proinflammatory cytokines were unaffected in macrophages from TLR4 deficient mice, suggesting that TLR4 signaling is not important for cytokine production in macrophages in this model. However, it is likely that the induction of proinflammatory cytokine production and activation of innate immune response following *T. congolense* infection may involve interactions between several TLRs that signal via MyD88. Further studies are required to clearly delineate the key parasite molecules involved in TLR2-MyD88 dependent resistance to *T. congolense* infection.

In conclusion, our studies identify for the first time TLR2- and MyD88-dependent activation of MAPKs and STATs as key intracellular events that are involved in cytokine production and enhanced resistance *T. congolense* infection in mice. Deficiency of TLR2 leads to uncontrolled first wave of parasitemia and acute death in an otherwise relatively resistance C57BL/6 mice. Understanding the receptors, adaptor proteins and the complex signaling pathways involved in immunity to African trypanosomiasis is of great interest and could eventually be used to develop novel strategies to enhance protective immunity and prophylaxis against the infection.

## Data Availability Statement

The raw data supporting the conclusions of this manuscript will be made available by the authors, without undue reservation, to any qualified researcher.

## Ethics Statement

The animal study was reviewed and approved by the University of Manitoba Animal Care Committee and experiments were carried out in accordance with the regulation of the Canadian Council on Animal Care.

## Author Contributions

SK, CO, RS, FO-A, PJ, and JU: design, acquisition, analysis, and interpretation of data. SK and CO: drafting. SK, CO, and JU: critical review. All authors reviewed and approved the manuscript for publication.

### Conflict of Interest

The authors declare that the research was conducted in the absence of any commercial or financial relationships that could be construed as a potential conflict of interest.
