# Supplementary material for: TLR-2 and MyD88-Dependent Activation of MAPK and STAT Proteins Regulates Proinflammatory Cytokine Response and Immunity to Experimental Trypanosoma congolense Infection
Source: Front Immunol. 2019 Nov 22;10:2673. doi: 10.3389/fimmu.2019.02673 (PMC6883972; doi:10.3389/fimmu.2019.02673)
Supplement: Supplementary file 1 [file Data_Sheet_1.pdf]

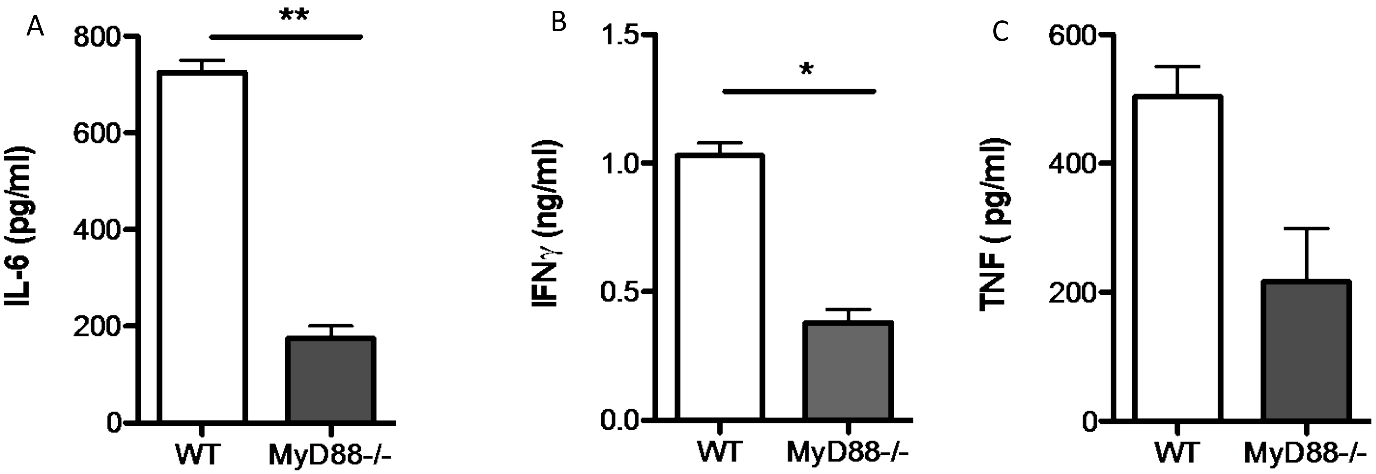

Supplementary Figure 1: Lower levels of proinflammatory cytokines in serum of *T. congolense*-infected MyD88$^{-/-}$ mice. IL-6 (A), IFN-γ (B) and TNF (C) levels in serum of infected (day 9 post-infection) WT and MyD88$^{-/-}$ mice were determined by sandwich ELISA. The data presented are representative of 2 independent experiments with similar results. *, $p < 0.05$; **, $p < 0.01$.

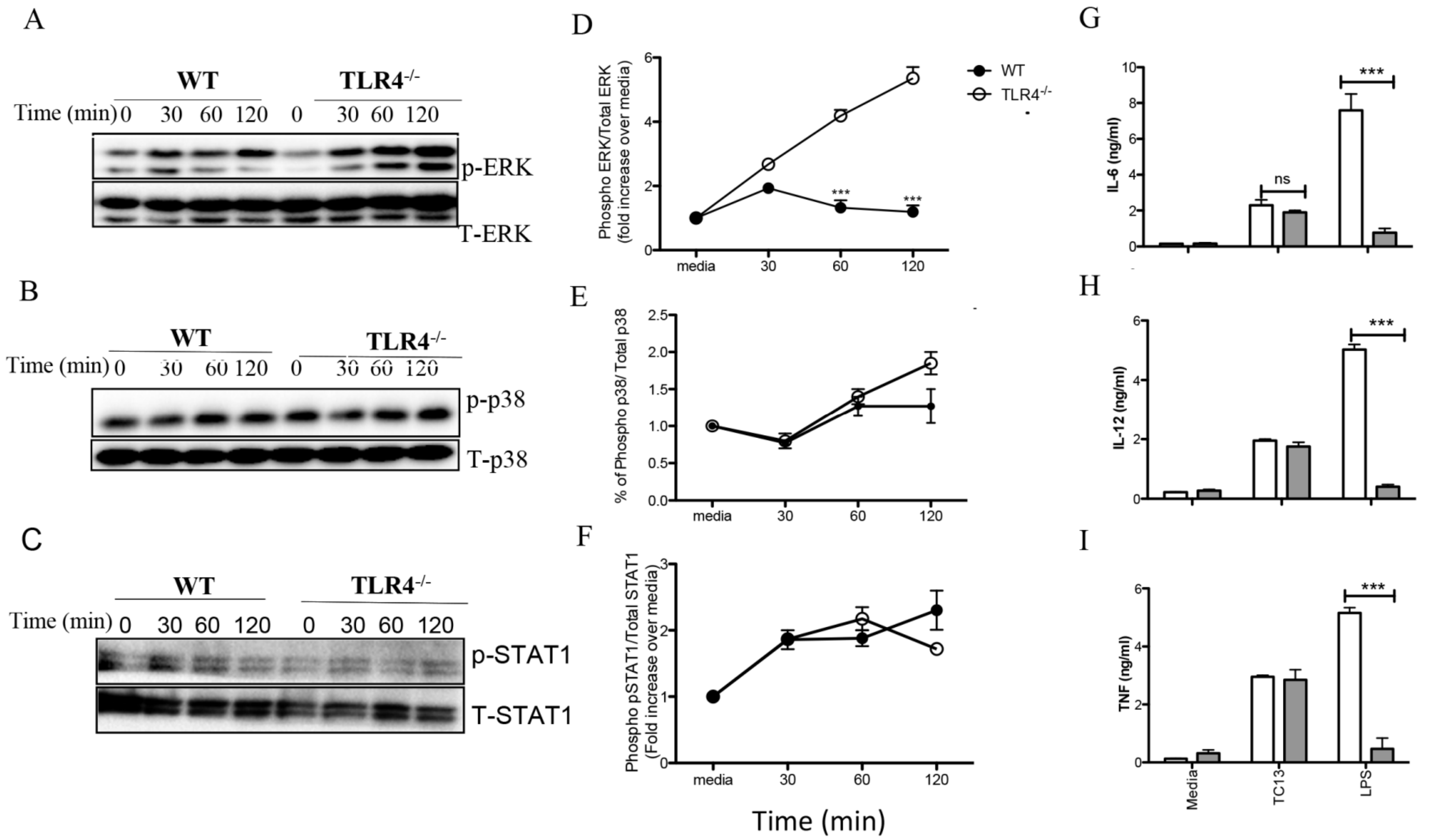

Supplementary Figure 2. TLR4 is not required for *T. congolense*-induced MAPK and STAT phosphorylation and cytokine production in macrophages. BMDMs were differentiated from WT and TLR4$^{-/-}$ mice and stimulated with WCE. At the indicated times, the cells were lysed and lysates were assessed by western blot for phosphorylation of ERK (A), p38 (B) and STAT1 (C) using appropriate primary and secondary antibodies. The same blots were stripped and re-probed with antibodies against total ERK, p38 and STAT1 and used as loading controls. The ratios of phosphorylated ERK (D), p38 (E) and STAT1 (F) to their respective total proteins were calculated by densitometry and plotted as line graphs (D-F). Some cells were stimulated with WCE or LPS for 24 hr. and the levels of IL-6 (G), IL-12 (H) and TNF (I) in the culture supernatant fluids were determined by ELISA. The data presented are representative of two independent experiments with similar results. *, $p < 0.05$; **, $p < 0.01$; ***, $p < 0.001$. ns; not significant.

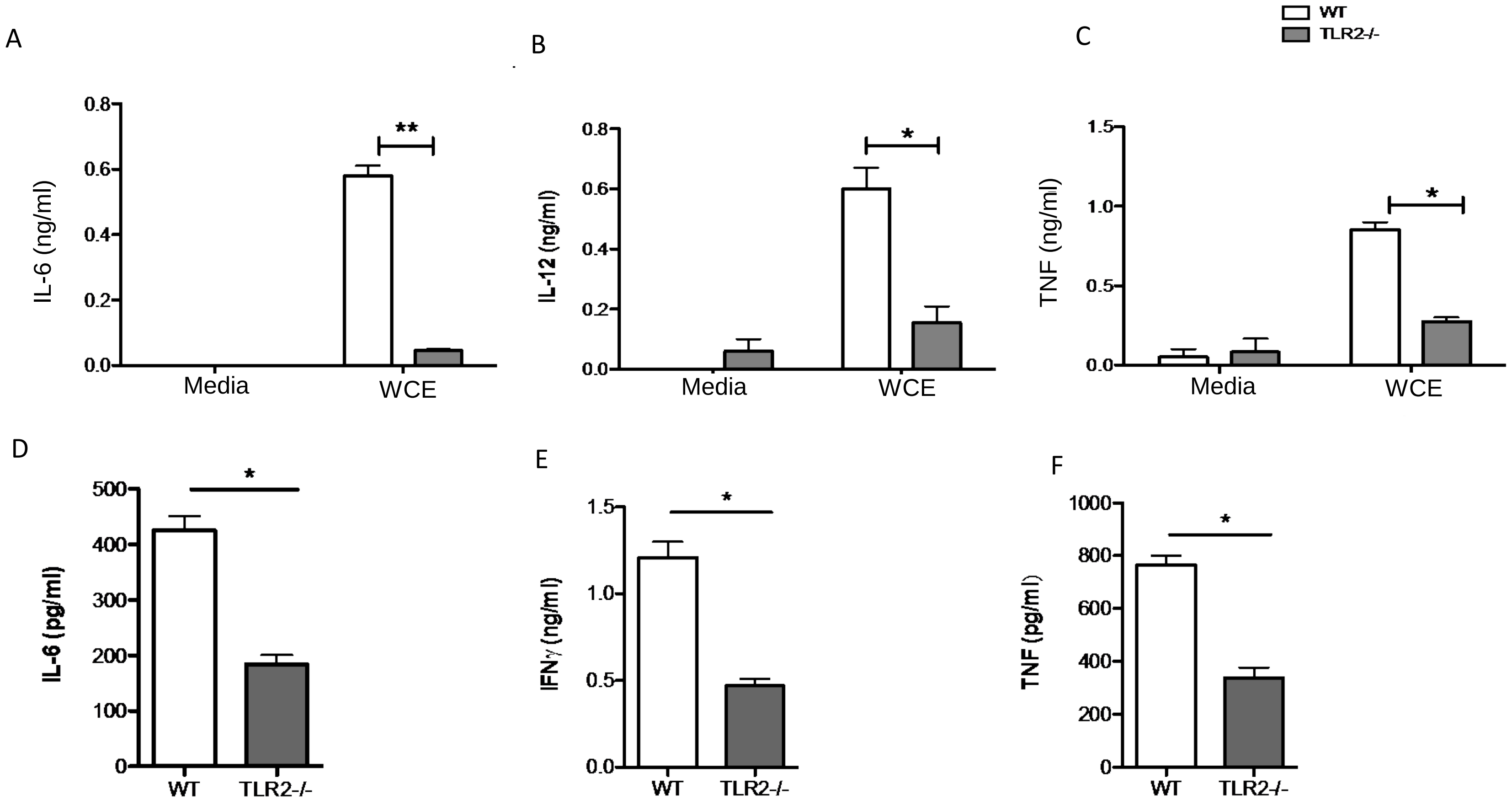

Supplementary Figure 3: Deficiency of TLR2 impairs *T. congolense*-induced proinflammatory cytokine response. (A-C). Impaired IL-6, IL-12, and TNF production by TLR2$^{-/-}$ macrophages stimulated with WCE. BMDMs from WT and TLR2$^{-/-}$ mice were stimulated with WCE and after 24 hr the levels of IL-6, IL-12 and TNF in the supernatant fluids were measured by ELISA. (D-F) Lower levels of proinflammatory cytokines in serum of *T. congolense*-infected TLR2$^{-/-}$ mice. IL-6 (D), IFN-γ (E) and TNF-α (C) levels in serum of infected (Day 9 post-infection) WT and TLR2$^{-/-}$ mice were determined by sandwich ELISA. The data presented are representative of 3 (A-C) and 2 (D-F) independent experiments with similar results. *, $p < 0.05$; **, $p < 0.01$.

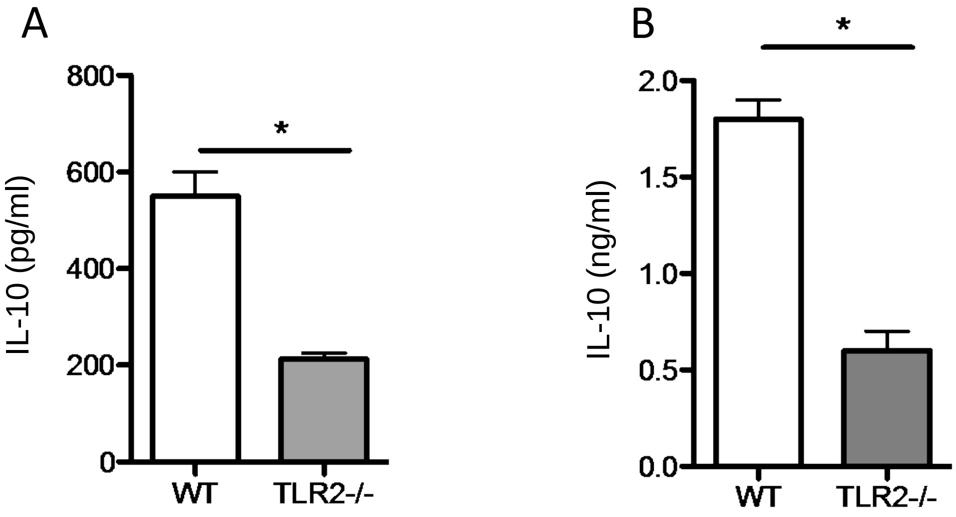

Supplementary Figure 4: Lower levels of IL-10 in *T. congolense*-infected TLR2$^{-/-}$ mice. Infected WT and TLR2$^{-/-}$ mice were sacrificed on Day 9 post-infection and the levels of IL-10 in the serum (A) or culture supernatant fluids of spleen cells (B) were determined by ELISA. The data presented are representative of 2 independent experiments with similar results. . *, $p < 0.05$.
